# Subjective Face Recognition Ability Is Linked to Objective Face Memory and Face Authenticity Judgment: Validation of the Traditional Chinese Version of the 20-Item Prosopagnosia Index

**DOI:** 10.3390/brainsci15111186

**Published:** 2025-10-31

**Authors:** Hai-Ting Wang, Kai-Mon Chuang, Taniya Rawat, Jia-Ling Lyu, Majeed Ali, Sarina Hui-Lin Chien

**Affiliations:** 1 Department of Biological Science and Technology, China Medical University, Taichung 404328, Taiwan; drwanghaiting88@gmail.com (H.-T.W.); taniya19971031@gmail.com (T.R.); 2 Department of Traditional Chinese Medicine, China Medical University, Taichung 404328, Taiwan; u108022419@cmu.edu.tw; 3 Graduate Institute of Biomedical Sciences, China Medical University, Taichung 404328, Taiwan; u105306601@cmu.edu.tw (J.-L.L.); majeedalipcr3@gmail.com (M.A.); 4 Neuroscience and Brain Disease Center, China Medical University, Taichung 404328, Taiwan

**Keywords:** developmental prosopagnosia, face blindness, face perception, PI-20, CFMT

## Abstract

**Background/Objectives:** Developmental prosopagnosia (DP) is a neurodevelopmental disorder marked by lifelong face recognition difficulties. The 20-item Prosopagnosia Index (PI-20) has become an important tool for screening individuals with DP and has been validated across various groups of English speakers worldwide. Recently, other language versions of PI-20, such as a Simplified Chinese one, have been developed and validated. Given the significant differences between Simplified Chinese and Traditional Chinese, as well as the distinct user populations, this study aims to validate a Traditional Chinese adaptation of the PI-20 using a standardized face memory task and a novel face authenticity judgment task in Mandarin-speaking populations. **Methods and Results:** In Study 1 (*n* = 94) and Study 2 (*n* = 138), we tested two large independent samples of college students using the English PI-20 and the Traditional Chinese PI-20, respectively. The results show strong internal consistency and similar score distributions in both versions of the PI-20. In Study 3 (*n* = 64), we examined the correlation between PI-20 scores and performance on the Cambridge Face Memory Test (CFMT). We found a significant correlation with the CFMT score and the Traditional Chinese version, but not with the English version PI-20. In Study 4 (*n* = 32), we examined whether PI-20 scores correlated with a face authenticity judgment task where participants judged whether the face image was real or AI-synthesized. Results showed that PI-20 negatively correlated with accuracy in judging real faces, but not with judging AI-synthesized faces. **Conclusions:** Overall, this study shows that although Taiwanese participants validly respond to the original PI-20, the Traditional Chinese version exhibited a stronger association with their objective face memory skills and showed a link to participants’ knowledge about real faces, which is a new finding. The Traditional Chinese PI-20 can serve as a dependable and useful tool in future research.

## 1. Introduction

The ability to recognize faces is fundamental to daily social interactions and communication. Human beings show intrinsic preferences for face-like stimuli and are sensitive to the face configuration in the earliest stages of development [1,2,3,4,5,6,7]. Throughout a prolonged developmental period during childhood [8,9,10,11], the human brain establishes a highly sophisticated and mature face processing system [12], which enables rapid and effortless recognition of a person’s identity, gender, race, and emotional expressions. However, the ability to recognize newly encountered faces varies significantly among individuals, resulting in a wide spectrum of differences. On one end of the spectrum are the so-called “super-recognizers,” [13,14] who exhibit extraordinary abilities in face memory and perception and can even identify celebrities before they become famous. On the opposite end are individuals with developmental prosopagnosia, who experience significant and lifelong difficulties in recognizing faces encountered daily.

Developmental prosopagnosia (DP), also known as congenital prosopagnosia (CP), is a neurodevelopmental disorder characterized by lifelong severe difficulties recognizing faces with normal low-level vision and general cognitive abilities [15,16,17,18]. DP prevails among family members, and many pedigrees are drawn by interviewing cases of DP. This indicates that DP is highly heritable, with largely unexplored genetic components [16,19]. In contrast to the acquired prosopagnosia (AP), individuals with developmental prosopagnosia do not have a history of brain trauma or injury. In the past, several objective standardized measures are used to assess face recognition ability, including the memory-based tests such as Warrington Recognition Memory for Faces Test (RMF) [20], the Famous Faces Recognition Test (FFRT) [21], the Benton Facial Recognition Test (BFRT) [22], and the Cambridge Face Memory Test (CFMT) [23], as well as perception-based tests such as the Cambridge Face Perception Test (CFPT) [24] and the Glasgow Face Matching Test (GFMT) [25]. In addition, subjective self-report instruments also play an important role in helping the diagnosis of DP to better reflect the face recognition difficulties and self-awareness in daily life [26].

Shah et al. highlighted the importance of self-reporting and introduced the 20-item prosopagnosia Index (PI-20) as a short, validated self-report questionnaire designed for assessing traits relating to prosopagnosia. This questionnaire consists of 20 statements describing daily experiences in face recognition. Respondents evaluate each statement on a 5-point scale, ranging from strongly agree to strongly disagree, to indicate how much the statements reflect their experiences. Higher scores on the PI-20 indicate poor face recognition ability. Shah’s study confirmed strong correlations between PI-20 scores and objective face recognition tests. In contrast, no correlation between PI-20 scores and non-face object recognition was observed [27,28]. Since PI-20 was published, it has become one of the most widely used self-report instruments for measuring face identification problems. Gray et al. confirmed a significant correlation between the original PI-20 and the Cambridge Face Memory Test (CFMT) in the general population, regardless of whether individuals administered PI-20 before or after the feedback from the CFMT [29]. Moreover, when grouping respondents according to their PI-20 scores, both the low- and the high-PI-20 score groups moderately correlated with the performance of CFMT. Compared to the low-PI-20 score group, the high-PI-20 score group achieved significantly lower CFMT scores. Similar results were obtained with the Australian version of CFMT (CFMT-A) [30]. These findings collectively confirm that people have the necessary insight into their face recognition ability.

Given the importance of face recognition in daily life, the PI-20 has been translated into different language versions for lab research and application in the general population. The significant negative correlations between PI-20 and CFMT have been replicated in various PI-20 versions, including those in Portuguese, Polish, Danish, Italian, Japanese, French, and Mexican Spanish [31,32,33,34,35,36,37]. Table 1 summarizes a list of recent publications examining the reliability and validity of the original English version of the PI-20 and its translated versions. To date, two studies using Simplified Chinese versions of PI-20 have been published. A moderate negative correlation was observed between PI-20 scores and the performance of the CFMT-Chinese, mainly driven by people at the lower and upper part of face recognition ability distribution [38]. Additionally, another study validated the Simplified Chinese versions of PI-20 by reporting a positive correlation with the adult autism quotient scale and a negative correlation with the empathy quotient [39].

Traditional Chinese, also known as complicated Chinese, has been used since the second century AD. It is primarily a logographical language in which the characters (and the parts of characters) are rich in meanings. Considering the substantial differences between Simplified Chinese and Traditional Chinese, as well as the divergent user populations, we recognize the need to translate the original PI-20 into a Traditional Chinese version. This translation will be validated with the language-specific population to ensure accuracy and cultural relevance.

The PI-20 has been used as a reliable tool to assess identity-specific face perception in DP. However, faces convey not only identity but also non-identity information, such as a person’s emotional expressions, age, and gender. Some studies suggested that the identity and non-identity face processing might partially share the perceptual processes in typical individuals [40]. From this perspective, people who have difficulties in identifying faces might also be impaired in non-identity face perception. Recently, Bennetts et al. [41] used a battery of tasks to assess non-identity face processing abilities such as emotional expressions, age, and gender perception in DP. They reported that the performance on the Philadelphia Face Perception Battery (PFPB) [42] gender task significantly predicted DP group classification and CFMT scores. These observations show that individuals with DP struggle with recognizing gender, which supports the idea that they also have difficulties with non-identity face perception [41]. Another example is the association between autistic traits and PI-20 score. Recently, several studies revealed that neurotypical adults who exhibited higher autistic traits also reported a higher PI-20 score [39,43], indicating that higher levels of autistic traits are associated with greater self-reported difficulties in face recognition. Similarly, individuals with ASD tended to have higher self-reported PI-20 scores than age-matched neurotypical adults [44].

In recent years, there has been a growing interest in another aspect of non-identity face perception: face authenticity judgment. As artificial intelligence (AI) technology advances at an unprecedented rate, synthesized faces exhibit such hyper-realism that distinguishing between real and AI-synthesized faces has become increasingly challenging. Nightingale & Fared [45] evaluated the photorealism of AI-synthesized faces by using face authenticity tests where participants were asked to classify faces as real or synthesized. In their Experiment 1, the mean accuracy of a large sample of adults (*n* = 315) was 48.2%, close to the chance level of 50%. In Experiment 2 (*n* = 219), where training and trial-by-trial feedback were provided, the mean accuracy improved to 59.0%; however, it remained only slightly above chance. Based on these results, they concluded that AI-synthesized faces were indistinguishable from real faces. Beyond their conclusion, we observed that the accuracy of each participant varied widely (i.e., ranging from 25% to 90% for Experiment 1 and 35% to 75% for Experiment 2). This suggests that there are large individual differences in detecting face authenticity in the general population, and to date, no studies have investigated these differences.

The present study aims to validate the Traditional Chinese version of PI-20 with a standardized objective face memory test and a novel face authenticity judgment. To this end, we conducted a series of four studies to examine the internal consistency, reliability, and construct validity of the Traditional Chinese version of PI-20. In Study 1, we assessed Taiwanese college students’ self-reported face recognition abilities using the original English version of PI-20. The goal was to evaluate whether the English version of the PI20 functions as a valid and reliable measure for individuals whose native language is not English but possess a certain level of English proficiency. Establishing this would provide an important basis for cross-cultural applications of the PI20 and for comparisons across populations with different linguistic backgrounds. Cronbach’s alpha and factor analysis were applied to evaluate its internal consistency and whether the test presents a single-factor structure. In Study 2, we assessed another sample of Taiwanese college students using the Traditional Chinese translated version of the PI-20. We compared the results obtained from both the original and the Traditional Chinese versions of the PI-20 to evaluate consistency in scores. Similarly, Cronbach’s alpha and factor analysis were applied to evaluate its internal consistency and whether the test presents a single-factor structure. Study 3 aims to test the content validity by examining the relationship between the Cambridge Face Memory Test (CFMT) and both the original PI-20 and its Traditional Chinese version individually. We intended to evaluate whether the findings observed in previous studies with the original PI-20 would extend to the newly translated version. We expected to find negative correlations between PI-20 scores and CFMT performance for both versions. We also wanted to compare the correlations from the original and Traditional Chinese versions to see if any significant differences appeared due to cultural or language factors. Study 4 serves as another test of content validity regarding non-identity face processing ability. We investigated the correlation between the Traditional Chinese version of PI20 and the performance of the Face Authenticity Judgment Task. We hypothesized that PI20 scores—reflecting self-reported difficulties with daily face recognition experiences—would be negatively correlated with accuracy in judging real faces.

## 2. Study 1

### 2.1. Participants

A sample of 94 undergraduate students (45 males, 49 females) participated in the study. They were mostly freshmen and sophomores recruited from the Chinese Medical University, Taichung, Taiwan, and with Traditional Chinese as their first language and English as their second language. The average level of English proficiency in reading among university freshmen students in Taiwan is above B1 (based on the Common European Framework of Reference for Languages, CEFR). All participants had normal or corrected-to-normal vision (20/20). Informed consent was obtained from each participant before the study. The present study adhered to the ethics of scientific publication as detailed in the Ethical Principles of Psychologists and the Code of Conduct [46] and to the Committee on Publication Ethics (COPE) guidelines. The Institutional Review Board of the Research Ethics Committee approved the study protocol at China Medical University Hospital, Taichung, Taiwan (certificate number: CMUH110-REC2-260 (CR-2)).

### 2.2. Procedures

After signing the informed consent form, all participants self-administered the paper-and-pencil questionnaire of the original English version PI-20, with a slightly modified format for ease of answering (i.e., similar to the Italian Version [34]).

#### The Prosopagnosia-Index 20 Items (PI-20)

As mentioned above, the PI-20 is a self-report questionnaire developed by Shah et al. [27] to assess face recognition difficulties in individuals with developmental prosopagnosia. Based on a review of both qualitative and quantitative literature on DP, along with discussions with individuals affected by DP, Shah et al. [28] created 20 items describing experiences and challenges in recognizing faces in real life. Respondents indicate how much they agree with these statements about their experience on a 5-point scale, from strongly agree to strongly disagree. There are fifteen forward questions and five reverse questions (Items 8, 9, 13, 17, and 19). For the forward questions, each item is scored positively; “strongly agree” scores 5 points, while “strongly disagree” scores 1 point. For the five reverse questions, each item is scored conversely, where “strongly agree” scores 1 point, and “strongly disagree” scores 5 points. The total score was calculated by summing the points obtained from each item, which range from 20 to 100. Higher scores indicate more self-reported problems with face recognition. A score over 65 is considered high and may suggest (but does not necessarily confirm) developmental prosopagnosia [27,28].

### 2.3. Results

**Figure 1:**
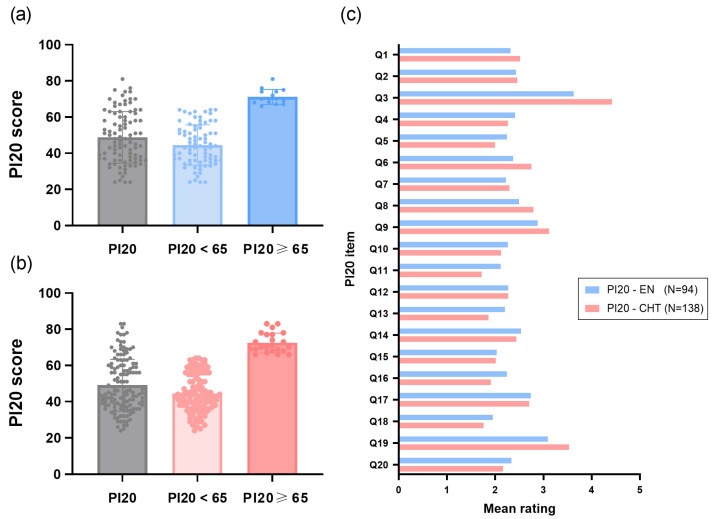
(**a**) The results of Study 1 showing the mean scores for all participants, those who scored below 65 (PI-20 < 65), and those who scored above 65 (PI-20 ≥ 65). Each dot represents an individual’s score. (**b**) The results of Study 2 showing the mean scores for all participants, for those who scored below 65 (PI-20 < 65), and those who scored above 65 (PI-20 ≥ 65). Each dot represents an individual’s score. (**c**) The mean responses across participants for each item in Study 1 (light blue bars) and Study 2 (pink bars).

The total mean score of the English version of PI-20 was 48.76 (*SD* = 14.26). Of the 94 participants, 79 (39 males, 40 females) scored lower than 65, which was 84% of the sample. Fifteen participants (6 males, 9 females) scored equal to or higher than the cutoff score of 65, which was about 16%. Figure 1a shows the group mean scores and the individual data points for those scored lower than 65 and those higher than 65. The Chi-square test of independence indicated no significant association between participants’ gender and the proportion of those scoring higher than 65 (*χ*^2^ (1, 94) = 0.443, *p* = 0.506). We calculated the Cronbach’s α value for the English version of PI-20, which was 0.92, indicating that Taiwanese participants responded to the 20 items with a high internal consistency. Confirmatory factor analysis with Varimax rotation further supports good construct validity for Study 1. Using eigenvalue and the Scree plot (elbow) analysis, we confirmed that a strong single-factor structure (factor 1 with an eigenvalue of 9.0) accounts for 45.0% of the total variance.

## 3. Study 2

### 3.1. Participants

Another independent sample of 138 undergraduate students (53 males, 85 females) was recruited in the same way as in Study 1. They were mostly freshmen and sophomores recruited from China Medical University, Taichung, Taiwan, and with Traditional Chinese as their first language and English as their second language. All participants had normal or corrected-to-normal vision (20/20). Informed consent was obtained from each participant before the study. The present study adhered to the ethical principles of scientific publication as detailed in the Ethical Principles of Psychologists and Code of Conduct [46] and to the guidelines of the Committee on Publication Ethics (COPE). The Institutional Review Board of the Research Ethics Committee approved the study protocol at China Medical University Hospital, Taichung, Taiwan (certificate number: CMUH110-REC2-260 (CR-2)).

### 3.2. Procedures

The procedure was the same as in Study 1. All participants self-administered the paper-and-pencil questionnaire of the Traditional Chinese-translated version of PI-20, which had a similar portrait-layout format as in Study 1 (see Appendix A).

#### The Traditional Chinese Version of Prosopagnosia-Index 20 Items (PI-20)

In this study, we meticulously translated the original PI-20 into the Traditional Chinese version and conducted a validation process. Two native Taiwanese researchers who worked collaboratively on the forward translation [47,48] ensured that the nuances and cultural context were effectively captured. Several revisions were made to decrease differences between the original version and the Traditional Chinese adaptation. The final translation was determined based on semantic and conceptual equivalence. The same scoring scheme used in Study 1 was applied to the Traditional Chinese version.

### 3.3. Results

The total mean score of the Traditional Chinese version of PI-20 is 49.09 (*SD* = 14.31). Of the 138 participants, 115 (47 males, 68 females) scored lower than 65, which was 83.3% of the sample. Twenty-three participants (6 males, 17 females) scored equal to or higher than the cutoff score of 65, which was about 16.7% of the sample. Figure 1b shows the group mean scores and the individual data points for those scored lower than 65 and those higher than 65. Similarly, the Chi-square test of independence revealed no significant association between participants’ gender and the proportion of those scoring higher than 65 (*χ*^2^ (1, 138) = 1.771, *p* = 0.183). The Cronbach’s α value for the Traditional Chinese version of PI-20 was 0.93, indicating that Taiwanese participants responded to these Traditional Chinese-translated 20 items with a high internal consistency. To test whether this sample of Taiwanese adults who received the Traditional Chinese PI-20 (Study 2) scored differently from those who received the English version PI-20 (Study 1, mean score = 48.76, (*SD* = 14.26)), we conducted an independent sample *t*-test. There was no significant difference between the two samples (*t* (230) = 0.174, *p* = 0.862). Confirmatory factor analysis with Varimax rotation further supports good construct validity for Study 2. Using eigenvalue and the Scree plot (elbow) analysis, we confirmed that a strong single-factor structure (factor 1 with an eigenvalue of 6.18) accounts for 30.92% of the total variance.

## 4. Study 3

### 4.1. Participants

In Study 3, we recruited two additional groups of well-matched participants, matched for gender and age. Each group had 32 adults, half male and half female. The Group 1 (age (*M* ± *SD*) = 23.65 ± 3.7 yrs; 16 females, age (*M* ± *SD*) = 23 ± 4.4; 16 males, age (*M* ± *SD*) = 24 ± 3.2) completed the original English version PI-20 and the CFMT, while Group 2 (age (*M* ± *SD*) = 23.25 ± 3.62 yrs; 16 females, age (*M* ± *SD*) = 23 ± 4.5; 16 males, age (*M* ± *SD*) = 23 ±2.8) completed the Traditional Chinese version of PI-20 and CFMT. The participants in this Study 3 were not involved in either Study 1 or Study 2. Informed consent was obtained from each participant before the study. The present study adhered to the ethical principles of scientific publication as detailed in the Ethical Principles of Psychologists and Code of Conduct [46] and to the guidelines of the Committee on Publication Ethics (COPE). The Institutional Review Board of the Research Ethics Committee at China Medical University Hospital, Taichung, Taiwan, approved the study protocol (certificate number: CMUH110-REC2-260 (CR-2)).

### 4.2. Apparatus and Procedure

A desktop computer (Lemel LX3-TKBJ19-4S50A) with a 22-in monitor (Acer P221W) was used to run the on-line CFMT. The order of the tests was counterbalanced: half of the participants completed PI-20 followed by the CFMT, while the other half completed the tests in the reverse order. Most participants completed the English PI-20 or the Traditional Chinese PI-20 with a paper-and-pencil questionnaire.

#### The Cambridge Face Memory Test (CFMT)

The Cambridge memory test is a standardized face memory test published by Duchaine & Nakayama (2006) [23]. Previous studies have consistently shown that the score of CFMT negatively correlated with the score of PI-20, providing a complementary evaluation of face recognition. In this study, we applied the CFMT to validate both versions of the PI-20. The online test of CFMT consists of four stages: practice, introduction/same images, novel images, and novel images with noise. During the practice stage, participants familiarized themselves with the procedure using cartoon faces. Later, they were introduced to six target faces and tested with forced-choice items featuring three faces with increasing difficulties. There were a total of 72 trial items. The total number of correctly answered trials was transformed into a percentage. The average score on this test is around 80% correct for adults. A score of 60% or below may indicate face blindness.

### 4.3. Results

**Figure 2:**
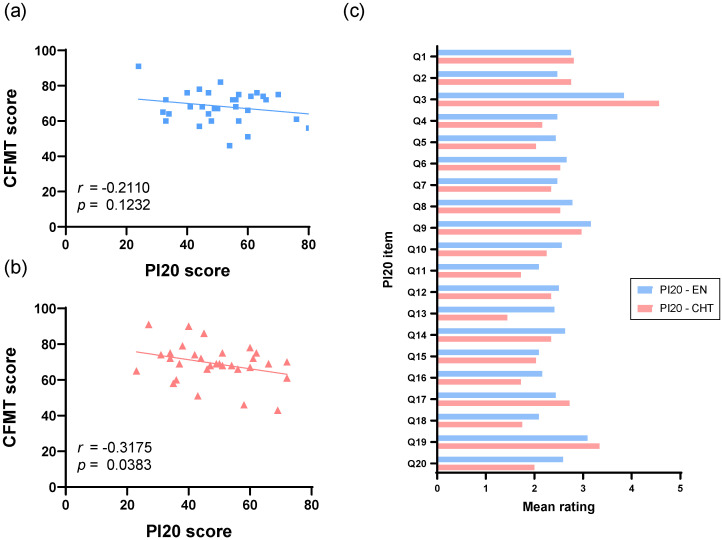
(**a**) The correlation between the English version of PI-20 scores and the CFMT scores in Group 1. (**b**) The correlation between the Traditional Chinese version of PI-20 scores and the CFMT scores in Group 2. (**c**) The mean responses for each item of the PI-20 questionnaire for both versions in Study 3.

For Group 1, the mean score of the original English version PI-20 was 51.69 (*SD* = 13.00). Of the 32 participants, 5 (15.6% of the sample) scored equal to or higher than 65. For Group 2, the mean score of the Traditional Chinese version PI-20 was 48.34 (*SD* = 13.03). Of the 32 participants, 4 (12.5% of the sample) scored equal to or higher than 65. An independent two-sample *t*-test revealed no significant difference between the two group mean scores (*t* (62) = 1.028, *p* = 0.308). For CFMT, the total number of correctly answered trials (out of 72) was transformed into a percentage. The mean percentages for Group 1 and Group 2 were 68.28 (*SD* = 9.14) and 69.19 (*SD* = 10.50), respectively, which were not significantly different (*t* (62) = 0.368, *p* = 0.714). Correlational analyses further revealed that the English PI-20 (*r* = −0.211, *p* = 0.123) and the Traditional Chinese version of PI-20 (*r* = −0.317, *p* = 0.038) both showed negative correlations with participants’ CFMT scores. Importantly, only the correlation strength for the latter reached statistical significance. Figure 2 illustrates the correlational scatter plots of PI-20 and CFMT scores.

## 5. Study 4

### 5.1. Participants

A total of 32 Taiwanese adults (16 males and 16 females, with a mean age (*M* ± *SD*) of 22.54 ± 1.94 years) participated in the study and were included in the final sample. The participants were primarily undergraduate or graduate students from China Medical University, Taiwan. To control for previous exposure to individuals of other races, participants were pre-screened for their cultural experiences; those who had lived in countries with a predominantly Caucasian population for more than three months were excluded from recruitment. The participants in Study 4 were not involved in previous studies. Informed consent was obtained from each participant before the study. The present study adhered to the ethical principles of scientific publication as detailed in the Ethical Principles of Psychologists and Code of Conduct [46] and to the guidelines of the Committee on Publication Ethics (COPE). The Institutional Review Board of the Research Ethics Committee approved the study protocol at China Medical University Hospital, Taichung, Taiwan (Certificate number: CMUH110-REC2-260 (CR-3)). An additional participant (female, age 23) was tested but excluded from the final data set due to an extremely high PI-20 score (89).

### 5.2. Apparatus and Stimuli

A desktop computer Lemel LX3-TKBJ19-4S50A (Lemel-Synnex Technology International Corp., Taipei, Taiwan) with a 22-in monitor Acer P221W (Acer Inc., New Taipei City, Taiwan) and E-Prime 2.0 (Psychology Software Tools, Sharpsburg, PA, USA) was used to run the task and record participants’ responses. The face stimuli were adapted from the stimulus set developed by Nightingale and Farid (2022) [45], which contains both real and AI-synthesized faces generated using StyleGAN2 (Karras et al., 2020) [49]. For each synthesized face, a matching real face from the underlying StyleGAN2 training database, aligned by gender, age, race, and overall appearance, was selected. The face stimulus set included faces across different genders, four races/ethnicities (Black, East Asian, South Asian, and White), and a wide range of estimated ages from children to older adults, with each set controlled such that the paired faces shared the same gender, race, and hairstyle. The size of each image on the screen measured approximately 13.5 cm high and 13.5 cm wide.

### 5.3. Procedures

The study was conducted in a quiet room. At the beginning, participants viewed a short introductory video explaining how AI technology can generate realistic human faces and completed the Traditional Chinese version of the 20-item Prosopagnosia Index (PI20), which assessed individual differences in self-reported face recognition ability. To avoid possible response bias from reflections based on self-perceived performance in the authenticity judgment, in most cases, participants completed the paper-and-pencil questionnaire before the computerized face authenticity judgment task.

Participants sat approximately 57 cm from the monitor, with their chairs adjusted to ensure their eyes were aligned with the centre of the screen. Curtains were closed on both sides to minimize visual distractions. Instructions for the face authenticity judgement appeared on the screen at the start of the task, indicating that images would be displayed one at a time and that each should be classified as either real (“1”) or synthetic (“3”). Participants initiated the practice session by pressing the space bar after reviewing the instructions. Each trial began with a fixation cross displayed for one second, followed by the presentation of a face image; this fixation–image sequence continued throughout the task. The practice session comprised 16 trials (4 race/ethnicity × 2 sex × 2 types (real and synthetic)) to familiarize participants with the procedure before the formal task. The formal session consisted of 96 trials, drawn from eight predefined image sets (4 race/ethnicity × 2 sex × 2 types × 6 selected sets). Within the formal experimental block, images were presented in a randomized order without replacement to prevent repetition. Both practice and formal sessions commenced with a space bar press, after which the experimenter left the room to avoid introducing additional distractions. Response accuracy and response times were recorded for every trial.

### 5.4. Results

The total mean score of the Traditional Chinese version of PI-20 is 44.75 (*SD* = 12.56). Of the 32 participants, 30 participants (15 males, 15 females) scored lower than 65, which was 93.75% of the sample. Two participants (one male, one female) scored equal to or higher than the cutoff score of 65, which was about 6.25%.

#### 5.4.1. Performance in Face Authenticity Judgment

We conducted a 2-way repeated measure ANOVA on the accuracy of face authenticity judgment, with Face Type (Real, Synthetic) and Race (Black, East Asian, South Asian, White) as the within-subject factors. The main effect of Face Type was not significant (*p* = 0.113); the mean accuracy for real and synthetic was *M* = 0.554. (*SE* = 0.028) and *M* = 0.475 (*SE* = 0.034), respectively. One-sample *t*-tests against chance level (50%) showed that accuracy for real faces was significantly above chance (*t* (31) = 1.930, *p* = 0.031), but accuracy for synthetic faces did not differ from chance (*t* (31) = −0.702, *p* = 0.756). The main effect of Race was significant (*F* (3, 93) = 3.725, *p* = 0.015, η_p_^2^ = 0.107). From high to low, the mean accuracies for recognizing South Asian, East Asian, Black, and White faces were *M* = 0.550 (*SE* = 0.026), *M* = 0.533 (*SE* = 0.028), *M* = 0.504 (*SE* = 0.021), and *M* = 0.473 (*SE* = 0.021), respectively. Post hoc comparisons (with an adjusted α level = 0.05/6 = 0.008) revealed that only the difference between White and South Asian faces reached statistical significance (*p* = 0.001). The remaining pairwise comparisons were not statistically significant.

#### 5.4.2. PI-20 Scores and Their Correlations with Facial Authenticity Judgments

**Figure 3:**
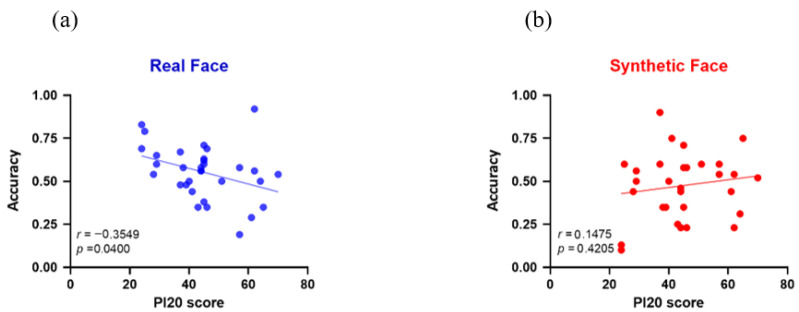
The correlations between the Traditional Chinese version of PI20 score and the accuracy of the Authenticity Judgment task for real faces (**a**) and synthetic faces (**b**).

Correlational analyses further revealed that PI-20 scores were significantly correlated with authenticity judgments (across all four races) of real faces (*r* = −0.365, *p* = 0.040), indicating that individuals with fewer self-reported face-recognition difficulties (lower PI-20 scores) were more accurate in identifying real human faces. When considering race-specific correlations, the strongest negative correlation was found for East Asian faces (*r* = −0.322, *p* = 0.072), followed by South Asian faces (*r* = −0.295, *p* = 0.101), Black faces (*r* = −0.275, *p* = 0.128), and White faces (*r* = −0.177, *p* = 0.333). This pattern suggests that participants who reported better face-recognition ability (i.e., lower PI-20 scores) tended to perform more accurately across all races, but particularly with East Asian faces, which is consistent with an own-race advantage. In contrast, correlations between PI-20 scores and judgments of synthetic faces were weak and non-significant (*r* = 0.147, *p* = 0.421). Figure 3 illustrates the correlations between the Traditional Chinese version of PI20 score and the accuracy of the Authenticity Judgment task for real and synthetic faces. Table 2 summarizes the mean accuracies for both real and synthetic faces in the Authenticity Judgement task and their correlations with PI-20 scores. 

## 6. Discussion

Owing to the substantial differences between the Simplified Chinese and Traditional Chinese and the distinct user populations that each script serves, we recognize the need to translate the original PI-20 into a Traditional Chinese version. This adaptation will ensure that the instrument is culturally and linguistically appropriate for Mandarin-speaking individuals in Taiwan, primarily targeting those who may experience difficulties with faces. The present study aims to address this need; we translated the original English PI-20 into a Traditional Chinese adaptation. Our translation process carefully considered the linguistic structure, cultural context, and idiomatic expressions unique to Traditional Chinese speakers. We explored the utility, reliability, and validity of the Traditional Chinese version in samples from a Mandarin-speaking population. We tested participants’ insight into their self-reported face recognition abilities with the English PI-20 (Study 1) and the Traditional Chinese PI-20 (Study 2). In Studies 1 and 2, Cronbach’s alpha and confirmatory factor analysis were applied to both versions of the PI-20 to evaluate its internal consistency and whether the test presents a single-factor structure. We also explored the content validity by examining the relationship between the Traditional Chinese PI-20, the original English PI-20, and the Cambridge Face Memory test (CFMT) (Study 3). Moreover, we investigated the correlation between the Traditional Chinese PI-20 and the performance of the Face Authenticity Judgment Task.

In Studies 1 and 2, two independent samples of young college students exhibited a similar mean PI-20 score and distribution. In Study 1, the mean score of the English PI-20 was 48.76 ± 14.26, with the mean score 44.51 ± 11.17 for low-PI-20 group (PI-20 < 65) and 71.13 ± 4.16 for high-PI-20 group (PI-20 ≥ 65), and with a high internal consistency (Cronbach’s α = 0.92). The high-PI-20 group on average scored more than three standard deviations above the low-PI-20 group, suggesting the English version of PI-20 distinguishes suspected prosopagnosia from typically developed controls. The mean score falls within the range of previous studies that also tested young adults of a similar age range such as Gray et al. (ranging from 40.10 ± 9.58 to 41.7 ± 10.10) [29], and Tsantani et al. (low-PI-20 group = 43.43 ± 9.08, high-PI-20 group = 77.04 ± 7.49) [30]. These results indicate that the original English PI-20 might be effective in a highly homogeneous sample, which consists of well-educated students from medical school, for self-reporting their difficulties in recognizing faces. Although English is the most commonly used language around the world, many people in Taiwan may not have strong English skills. Since PI-20 has been well-developed and widely used, we aim to eliminate the language barrier that might hinder its applications in the prosopagnosia research in Taiwan. This motivates us to translate and validate the Traditional Chinese PI-20.

In Study 2, the mean score of the Traditional Chinese PI-20 was reported as 49.09 ± 14.3. The mean PI-20 score for the low-PI-20 group (PI-20 < 65) was 44.42 ± 10.42, while the high-PI-20 group (PI-20 ≥ 65) had a mean score of 72.43 ± 5.44, showing similar subgroup means and proportions of low-PI-20 and high-PI-20 groups as observed in Study 1. The high-PI-20 group, on average, scored more than three standard deviations above the low-PI-20 group, suggesting the Traditional Chinese PI-20 can distinguish suspected prosopagnosia from typically developed controls. The translated 20 items have a good internal consistency (Cronbach’s α = 0.93), and the mean score is comparable to the Simplified Chinese PI-20 (47.89 ± 11.78) [38]. Based on these results, both the English PI-20 and the Traditional Chinese PI-20 showed good reliability. In terms of participant’s gender ratio for the Studies 1 and 2, although the ratios of female to male was higher in Study 2 (Female to Male, 85:53) than in Study 1(Female to Male, 49:45), the Chi-square tests indicated there was no significant difference between the gender ratios in these two samples (*χ*^2^ = 2.054, *p* = 0.152), and no associations between the gender ratio and the proportion of those scoring higher than 65 were observed in Study 1 (*χ*^2^ (1, 94) = 0.443, *p* = 0.506) and Study 2 (*χ*^2^ (1, 138) = 1.771, *p* = 0.183). Additionally, confirmatory factor analyses in both Studies 1 and Study 2 further confirmed that a strong single-factor structure was present in both studies.

In Study 3, we investigated the correlation between the scores of the English PI-20 and the Traditional Chinese PI-20, and performance on the CFMT, which is recognized as one of the most commonly used objective measures for assessing face recognition abilities. We hypothesized that the PI-20 scores could serve as a reliable predictor of performance on the CFMT, aligning with findings from previous studies (see Table 1). To control for potential confounding variables, we recruited two groups of participants closely matched in age, gender, and sample size. This careful selection process was crucial for ensuring the validity of our results and providing a clearer understanding of how the PI-20 scores assessed with two language versions relate to face recognition performance as measured by the CFMT for the Taiwanese population. We found no significant difference between the mean scores of the English PI-20 and the Traditional Chinese PI-20. Importantly, our results confirm that the Traditional Chinese PI-20 exhibits a significantly moderate, negative correlation with the CFMT (*r* = −0.3175). This relationship is consistent with previous studies and suggests that the Traditional Chinese PI-20 has good validity. Additionally, a negative but non-significant correlation was observed between the scores of the original English PI-20 (*r* = −0.2110) and CFMT. This suggests that participants’ self-evaluations with the translated adaptation of PI-20 could more accurately reflect on their face recognition skills. We believe that the Traditional Chinese PI-20 can serve as a reliable and viable tool for the initial screening of individuals experiencing face recognition problems in both clinical settings and laboratory research with the Taiwanese population.

Across Studies 1 to 3, we observed a high proportion of participants who scored above the PI-20 cutoff (≥65) in our samples. Indeed, self-report measures like the PI-20 are prone to subjective influences, which may result from sample-specific traits (such as increased self-awareness or reporting tendencies among university students) or measurement and recruitment biases. We believe this high rate may be due to university students having greater self-awareness or a tendency to report their daily face recognition experiences more readily. Estudillo & Wong [38] tested Chinese ethnicity students in the UK and Malaysia, and Oishi et al. [35] tested Japanese students in Japan; both reported similar central tendencies and score distributions (see Table 1). Furthermore, although our results may reflect a high proportion of scores above 65, we consider our data reliable; the proportion of participants scoring above 65 was consistent across two independent samples using the Traditional Chinese version of the PI-20 (Studies 2 and 3). It was also similar between the two independent samples that completed the English version (Studies 1 and 3). When comparing the Chinese and English versions, the proportions of participants exceeding the cutoff did not significantly differ. Based on these observations, we believe that our data with university students’ samples are highly consistent. However, it remains an open question whether the present results can be generalized to the entire Taiwanese adult population, with a wider age range and varying levels of English proficiency.

In Study 4, we investigated Taiwanese participants’ ability to distinguish between real and synthetic faces across four racial groups and examined how self-reported face recognition ability (PI-20) related to the task performance. Overall, participants performed slightly above chance when judging real faces (*M* = 0.554), while their accuracy for synthetic faces was lower (*M* = 0.476). However, the difference between the two was not statistically significant. This suggests that participants were not reliably more accurate with real than with synthetic images, indicating that detecting AI-generated images remains a challenging task. The effect of race was also significant: participants showed the highest accuracy for South Asian (near-race) faces, followed by East Asian (own-race) faces, Black faces, and White faces. This pattern suggests that, although it is challenging to make a facial authenticity judgment overall, we observed a relative advantage for broadly defined Asian faces, particularly near-race South Asian and East Asian faces, compared to Black and White faces, consistent with both near-race and own-race considerations.

More importantly, correlational analyses highlighted individual differences. PI-20 scores showed a significant negative correlation with accuracy for real faces (*r* = −0.365, *p* = 0.040), indicating that participants with fewer self-reported difficulties in face recognition were more accurate at identifying real human faces, a moderate effect linking everyday face recognition ability to authenticity judgments. Items on the PI-20 were created based on a review of relevant qualitative and quantitative literature on DP and in-depth interviews with individuals with DP to understand how much the condition disrupts normal daily activities. Since our daily experience involves mostly real faces rather than artificially created ones, scores on the PI-20 may therefore better represent one’s ability to recognize faces in real-world situations. The results of Study 4 align with Miller et al.’s (2023) [50] evidence of AI hyperrealism, whereby synthetic faces can appear highly human-like, reducing the perceptual cues people typically rely on to judge authenticity.

In sum, Taiwanese participants showed modest success in distinguishing between real and synthetic faces, with performance varying by race and being correlated to self-reported face recognition ability. We believe that including Study 4 offers a new test of content validity related to non-identity face processing ability. The rise of AI-generated faces marks an important new research area. We believe that this study is among the first to demonstrate a link between self-reported face recognition ability and facial authenticity judgment. The findings underscore both the promise and the challenges of human detection of AI-generated content as visual realism continues to increase.

## 7. Conclusions, Limitation, and Future Work

In conclusion, in a series of four studies, we investigated the reliability and validity of the Traditional Chinese version of the PI-20, and the results demonstrated good internal reliability (Studies 1 and 2) and content validity (Study 3), suggesting that the Traditional Chinese version questionnaire can be a valuable tool for assessing face recognition abilities among populations using Traditional Chinese, particularly in Taiwan. One novelty of this study is the examination of the relationship between PI20 and the ability to judge facial authenticity (Study 4). Our findings indicate that participants with fewer self-reported difficulties in face recognition were more accurate at identifying real human faces, a moderate effect linking everyday face recognition ability to facial authenticity judgments.

The present study has several limitations that must be borne in mind. One major limitation is that we only adopted the memory-based CFMT task to assess unfamiliar face recognition ability. Other objective measures of face processing capacity should be included to further validate the results. Another limitation is that our sample mostly consists of university students. This group tends to be younger and possesses above-average proficiency in English. Age is known to influence face recognition performance, and higher English proficiency may facilitate their understanding of task instructions or questionnaire items. These characteristics may therefore limit the external validity of our findings, that is, the extent to which the results can be generalized to populations of different ages or with diverse English proficiency levels. Lastly, we are aware of the imperfections in our translation. Although the materials were carefully translated and reviewed for clarity, the absence of backward translation could have introduced subtle semantic deviations from the original English version, potentially affecting the equivalence of meaning across languages.

Our future work will involve conducting various tests, including the Cambridge Face Perception Test (CFPT) [24] and the Glasgow Face Matching Test (GFMT) [25] to expand its content validity, as well as the Cambridge Car Memory Test (CCMT) [51] to verify the discriminant validity. Furthermore, we observe that the mean PI-20 scores in Taiwanese participants (ranging from 48.34 to 51.69) are similar to those reported in some studies [32,35,36,38], while being higher than those initially reported in the original studies [27,28]. This hints that the suggested cutoff score in the Taiwanese population may need further exploration. Additionally, integrating AI with brain imaging techniques best suited for temporal processing, such as EEG and MEG, may enhance stimulus control and improve the precision of individual assessments beyond behavioral manifestation. We intend to combine them in future research to advance our understanding of individual differences in face processing.

## Figures and Tables

**Table 1:** Summary of the PI20 scores obtained by the English version and the translated versions in other languages (PI20 and CFMT (%) are reported as mean ± SD).

Study	PI-20 Language	Enrollment	Size	Age (yrs)	PI-20	CFMT (%)	Correlation
Shah et al. (2015) [27]	English	Study 1 Local participants	TD controls 242 (155 F)	29.8	38.90 ± 10.88	N/A	N/A
			Suspected DPs 77 (47 F)	43.0	82.01 ± 9.34	N/A	
		Study 2 Local participants	TD controls 18 (6 F)	43.5	41.67 ± 12.10	84.3 ± 9.9	N/A
			Suspected DPs 18 (6 F)	46.7	81.22 ± 9.47	54.33 ± 9.65	N/A
		Study 4 Local participants	TD controls 87 (57 F)	28.6	N/A	79.98 ± 13.00	*r* = −0.683, *p* < 0.001
			Suspected DPs 23 (8 F)	45.8	N/A	56.42 ± 10.04	
Shah et al. (2015) [28]	English	Local participants	190 (131 F)	27.72	41.99 ± 13.40	GFMT 85.00 ± 11.92	*r* = −0.49, *p* < 0.001
Gray et al. (2017) [29]	English	University students	142 (86 F)	29.23	40.10 ± 9.58	80.65 ± 12.79	*r* = −0.394, *p* < 0.001
		University students	283 (177 F)	26.64	41.70 ± 10.10	76.80 ± 12.90	*r* = −0.390, *p* < 0.001
Tsantani et al. (2021) [30]	English	Low-PI20 group Website	225 (128 F)	36.14	43.43 ± 9.08	74.55 ± 13.56 (CFMT)	*r* = −0.23, *p* < 0.0001
						76.10 ± 11.90 (CFMT-A)	*r* = −0.23, *p* < 0.0001
		High-PI20 group Website	159 (112 F)	37.73	77.04 ± 7.49	60.46 ± 12.38 (CFMT)	*r* = −0.21, *p* = 0.009
						65 ± 13.47 (CFMT-A)	*r* = −0.36, *p* < 0.0001
Ventura et al. (2018) [31]	Portuguese	University students	123 (108 F)	20.40	42.02 ± 9.26	86.20 ± 10.24	*r* = −0.43, *p* < 0.0001
Marschollek et al. (2019) [32]	Polish	Social media	1276 (840 F)	28.3	49.6 ± 18	80.69	*r* = −0.42, *p* < 0.001
Estudillo & Wong (2021) [38]	Simplified Chinese	University students	255 (188 F)	21	47.89 ± 11.78	78.42 ± 11.38 (CFMT-Chinese)	*r* = −0.35, *p* < 0.001
Sun et al. (2021) [39]	Simplified Chinese	N/A	647 (464 F)	22.11	Not reported	N/A	N/A
Nørkær et al. (2023) [33]	Danish	University students	119 (84 F)	N/A	40.18 ± 10.49	60.46 ± 7.80	*r* = −0.34, *p* < 0.001
Tagliente et al. (2023) [34]	Italian	Online or in Lab	553 (346 F)	27.25	40.59 ± 9.05 (Age < 35)	70.82 ± 12.36 (Age < 35)	*r* = −0.18, *p* < 0.001
					41.27 ± 10.07 (Age ≥ 35)	63.94 ± 11.37 (Age ≥ 35)	
Oishi et al. (2024) [35]	Japanese	University students	116 (75 F)	20.68	46.34 ± 11.45	74.03 ± 0.10	*r* = −0.28, *p* = 0.02
Nigrou et al. (2024) [36]	French	Social media or University intranet	216 (155 F)	40.54	48.9 ± 16.6	75.4 ± 13.3	*r* = −0.361, *p* < 0.001
Mejia et al. (2025) [37]	Mexico Spanish	N/A	333 (127 F)	28.58	43.68 ± 9.36	75.56 ± 13.68	*r* = −0.229, *p* < 0.001

CFMT: Cambridge Face Memory Test; CFMT-A: Australian version of Cambridge Face Memory Test; CFMT-Chinese: Chinese version of Cambridge Face Memory Test; GFMT: Glasgow Face Matching Test.

**Table 2:** Summary of the accuracies for the real and synthetic faces in the Authenticity Judgement task and their correlations with PI-20 scores.

	Real Face					Synthetic Face				
	Black	East Asian	South Asian	White	All	Black	East Asian	South Asian	White	All
Mean	0.526	0.516	0.628	0.492	0.554	0.482	0.497	0.471	0.453	0.476
*SE*	0.042	0.041	0.036	0.031	0.028	0.042	0.036	0.047	0.039	0.034
Min	0.08	0.17	0.25	0.17	0.19	0.00	0.17	0.00	0.17	0.10
Max	0.92	1.00	1.00	1.00	0.92	1.00	0.83	0.92	0.83	0.90
Correlation with PI-20	−0.275	−0.322	−0.295	−0.177	−0.365	0.200	0.164	0.064	0.073	0.147
*p*-value	0.128	0.072	0.101	0.333	0.040 *	0.274	0.369	0.730	0.690	0.421

* *p* < 0.05.

## Data Availability

The original datasets (as an Excel file) for the validation studies have been made publicly available and stored at Open Science Framework via https://osf.io/6sqge/ (accessed on 27 October 2025).

## Appendix A

**繁體中文版20項臉孔失認症指標**						
**Traditional Chinese Version of the 20-Item Prosopagnosia Index**						
	**項 目 (Items)**	**評分 (Rating)** **1: 非常不同意；5: 非常同意** **1: Strongly disagree; 5: Strongly agree**				
1	我的臉孔辨識能力比大多數人差。	1	2	3	4	5
2	我對於臉孔的記憶力總是很差。	1	2	3	4	5
3	我發現臉部有顯著特徵的人特別容易辨識。	1	2	3	4	5
4	我時常把以前見過的人為陌生人。	1	2	3	4	5
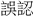
5	過去在學校裏，我要很費力才能認出同班同學。	1	2	3	4	5
6	當人們改變髮型或戴帽子，我要認出他們會有困難。	1	2	3	4	5
7	有時候我必須提醒剛認識的人，我的認臉能力很差。	1	2	3	4	5
8	我覺得在腦中描繪個別的臉孔是容易的。	1	2	3	4	5
9	我比大多數人更擅長叫出臉孔的名字。	1	2	3	4	5
10	如果沒有聽見某人的聲音，我就很難認出他們。	1	2	3	4	5
11	辨認臉孔的焦慮讓我想逃避某些社交或正式場合。	1	2	3	4	5
12	我必須比其他人更努力地去記住臉孔。	1	2	3	4	5
13	我非常有把握能從照片中認出我自己。	1	2	3	4	5
14	有時候我很難跟上電影情節是因為辨認角色有困難。	1	2	3	4	5
15	我的朋友和家人覺得我的認臉能力和臉孔記憶很差。	1	2	3	4	5
16	我感覺我常常會因為認不出別人而冒犯了他們。	1	2	3	4	5
17	即便是一群人在特定場合穿著相似的服裝 (例如：西裝、制服、泳裝)，我也能輕易地分辨每個人。	1	2	3	4	5
18	在家庭聚會中，有時候我會混淆家族成員。	1	2	3	4	5
19	我覺得認出名人在成名前的照片是容易的，即使他們的樣子已經改變很多。	1	2	3	4	5
20	我很難辨認出非平時情境下遇到的熟人 (例如：在購物時不預期地遇見同事)。	1	2	3	4	5
